# Technical Considerations and Avoiding Complications in Total Hip Arthroplasty

**DOI:** 10.5435/JAAOSGlobal-D-22-00234

**Published:** 2022-11-15

**Authors:** John W. Krumme, Sean Bonanni, Nirav K. Patel, Gregory J. Golladay

**Affiliations:** From the Department of Orthopaedic Surgery, University of Missouri–Kansas City, Kansas City, MO (Dr. Krumme and Dr. Bonanni), and the Department of Orthopaedic Surgery, Virginia Commonwealth University Medical Center, Richmond, VA (Dr. Patel and Dr. Golladay).

## Abstract

Total hip arthroplasty (THA) is considered to be the surgical procedure of the 20th century. Current projections show that by 2030, primary THA is expected to grow by 171%, with revision THA expected to increase by 142% in the same time frame. Although complications are not common, when they occur, they can cause notable morbidity to the patient. Understanding the unique anatomy and needs of each patient will prepare the surgeon to avoid soft-tissue or bony injury, optimize prosthesis placement, and decrease the risk of infection. This article aims to highlight common causes of early revision THA and provide specific technical strategies to avoid these complications. Following a systematic approach to the primary THA and using these techniques will assist the surgeon in avoiding complications to revision hip arthroplasty.

Total hip arthroplasty (THA) is one of the most successful procedures in the United States, with over 90% survivorship at 10 years and over 80% survivorship at 20 years.^1^ Current projections estimate that by 2030, primary THA is expected to grow by 171%.^1,2^ Similarly, revision THA is also expected to increase by 142%, and this trajectory will continue.^1^ Although complications are uncommon, when they occur, they cause notable morbidity to the patient.^2^ Common etiologies for failure include dislocation, infection, aseptic loosening, periprosthetic fracture, trunnion damage, and persistent pain.^3^ Early recognition of the potential intraoperative pitfalls and understanding how to avoid them help improve the final result.

There are multiple steps a surgeon can take to reduce the complication rate in THA. Preoperative templating and thorough medical review should be conducted with preoperative optimization and addressal of patient-specific anatomic considerations. Intraoperatively, optimizing exposure and hemostasis allow for optimal implant placement. Appropriate intraoperative checks can assist in the successful execution of the preoperative plan. We will discuss our systematic approach to avoid technical errors in a posterior approach THA, which may minimize indications for revision arthroplasty.

## Preoperative Assessment

Preoperative screening and preoperative optimization increase the chance of a successful surgery.^4^ Implementing standardized preoperative optimization protocols reduces 90-day cost of care to patients. Medical clearance from a primary care provider and any needed specialists reduces the risk of postoperative complications such as surgical site infections (SSIs), reduced length of stays, and rehospitalizations.^4,5^ Treating underlying malnutrition, attaining a hemoglobin A1c of less than 7.5%, treating anemia, and smoking cessation all can decrease length of stay, readmission, and total cost of care to each patients.^4^ Research has indicated that elevated hemoglobin A1c levels and hyperglycemia can predispose patients to prosthetic joint infections, with A1c levels more than 7.5 causing marked increases in the risk of these infections.^6,7^ Patients with controlled diabetes (hemoglobin A1c <7.5%) have similar rates of complications as those without diabetes.^6^ Follow-up and re-evaluation is needed to ensure that these modifiable risk factors are being treated appropriately.

**Figure 1:**
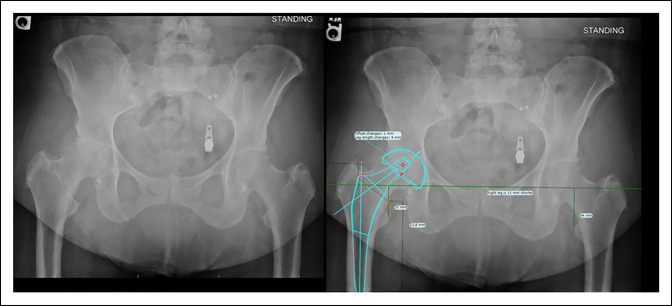
Template AP pelvic radiograph shows superior migration of the femoral head in (**A**), and the template in (**B**) shows the plan to restore the leg length and offset.

Technical preparation is important to avoid complications and provide a plan to restore leg lengths and offset as well as implant positioning.^7^ A thorough history and physical examination with radiographic templating (Figure 1) allows the surgeon to plan for unique aspects of the upcoming procedure. Planning for implant removal, deformity correction, the surgical approach, and ensuring the correct implants are available can be done before surgery to decrease surgical time and provide the most appropriate implant for the patient.^7^ An increase in surgical time can increase infection risk on an incremental basis.^8^ This imaging (Figure 1) allows the surgeon to assess leg lengths, offset, the quality of bone stock and morphology of the acetabulum, and the likely positioning of the implants and can usually predict the implant size within 1 to 2 increments.^5,7^

Preventing three common indications for revision arthroplasty (dislocation, aseptic loosening, and adverse local tissue reactions [ATLRs]) takes place preoperatively with templating, selecting appropriately sized implants, optimizing leg length and offset, and choosing appropriate bearing surfaces.

### Infection Prevention

Periprosthetic joint infection (PJI) prevention occurs in all facets of patient care. Addressing modifiable risk factors, perioperative intravenous antibiotic administration, limiting odds ratio traffic, reducing contamination, and sterile, silver-impregnated dressings all reduce the risk of PJI.

Modifiable risk factors should be addressed before surgery. Patients with hemoglobin A1c >7.5%, albumin <3.5 g/dL, and anemia <12 g/dL all increase the risk of PJI.^9^ Tobacco users have an increased risk of both wound complications and PJI when compared with ex-smokers and nonsmokers; however, there is no consensus on the interval between smoking cessation and surgery.^4,9,10^

Perioperative intravenous antibiotics administration before incision reduces the incidence of PJI.^11^ While draping, prepping the surgical site again before adhesive draping also reduces the risk of SSI, although evidence is not clear regarding prevention of PJI.^12^ Changing gloves after draping before handling surgical tools also reduces contamination.^11^ Body exhaust systems and surgical hood systems are thought to reduce the risk of PJI by controlling shedding.^11^ While body exhaust systems, which use a negative pressure system, reduce PJI, the surgical helmet systems have failed to show a statistically significant reduction in the risk of PJI.^13^ Recent literature has indicated that laminar airflow may not be as important in PJI prevention as previously considered. When patient factors are accounted for, laminar airflow does not provide a statistical improvement in PJI prevention.^14^ Conversely, limiting operating room traffic reduces the risk of SSIs.^11^ Allogeneic blood transfusion has also been associated with increased rates of PJI. Perioperative tranexamic acid and meticulous hemostasis limit perioperative blood loss and, thus, the need for blood transfusion.^11^ Ultimately, PJI prevention requires a comprehensive approach and appropriate steps to be taken before, during, and after total joint arthroplasty.

### Nerve Injury

In the posterior approach to the hip, the sciatic nerve and femoral nerve are at unique risk. Those with posttraumatic arthritis, dysplastic hips, and notable shortening are at increased risk of a nervous injury during THA.^15^

The sciatic nerve exits the greater sciatic notch and has a variable relative to the piriformis. The incidence of sciatic nerve injury during posterior THA is 0.068% to 1.9%.^15^ Injury can occur during the approach with retractor placement of the Charnley retractor or posterior capsular retractors. When placing posterior acetabular retraction, the sciatic nerve can run within 2 cm of the posterior acetabulum. Lengthening a chronically shortened limb >2 cm can also create a traction injury.^15^

The femoral nerve runs across the anterior hip, beneath the inguinal ligament. It is at greatest risk with anterior acetabular retractor placement. Errant anterior retractor placement can directly or indirectly compress the nerve as it crosses the hip joint. The incidence is 0.01% to 2.3%.^15^ Anterior retractor placement under direct visualization or with palpation of the anterior wall is recommended. Because the femoral nerve is at greater risk with inferior placement of this retractor, erring superior is recommended.^15^

### Abductor Injury

**Figure 2:**
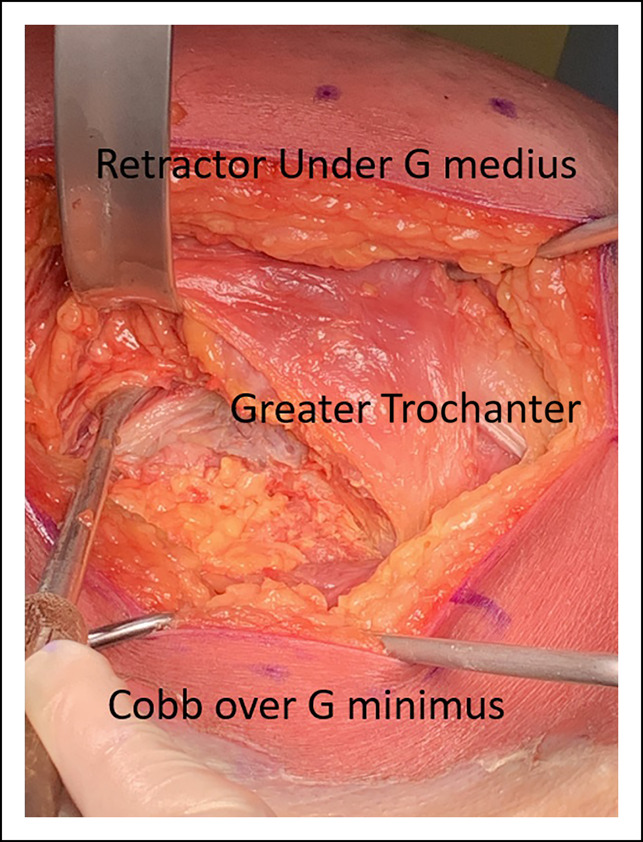
Photograph showing the approach to avoid abductor damage. The gluteus medius is protected by a blunt retractor while the Cobb elevator is developing the layer between gluteus minimus and capsule.

Perhaps the most important dynamic stabilizer of a THA is the gluteus medius and minimus muscles. Minimizing injury to these will improve stability of the prosthesis, help maintain normal gait mechanics, and prevent the incidence of heterotopic ossification (Figure 2).^16,17^ Damage to these muscles can also lead to deficiency and a cause of dislocation.^16^ Damage to the hip abductors classically has been associated with heterotopic ossification, which can also lead to abductor dysfunction and postoperative pain.^17^ Other approaches such as the direct lateral and anterolateral approaches do violate the abductor mechanism, which has been shown to cause gait changes and prolonged weakness of the abductor mechanism.^16^ In the posterior approach, we protect these muscles by identifying the posterior border of the gluteus medius and then protecting the tendon with a retractor carefully placed between the plane between the gluteus medius and minimus. Identification of the leading edge of the gluteus minimus is done, and it is dissected free of the capsule with the Cobb elevator avoiding damage to the muscle belly while avoiding injury to the muscle. The retractor between the lesser gluteals is then placed between capsule and gluteus minimus. We then conduct a reverse L-type capsulotomy to expose the hip joint.

### Cup Positioning

Cup positioning plays a vital role in the stability of THA. Keeping the arc of motion of the prosthetic joint within physiologic parameters is key to a stable prosthetic joint.^18^ Lewinnek described the safe zone for THA. Recent literature has questioned the importance of the Lewinnek safe zone because many of the THA dislocations occur with appropriately positioned acetabular implants.^18^ Since then, research has expanded to include a functional safe range of cup position based on dynamic spinopelvic motion.^19^ Intraoperative optimal cup positioning begins with patient positioning such as understanding where the patient's body is in relation to the table and floor is key in optimizing inclination and version and addressing any spinopelvic considerations. Once the acetabulum is exposed, removal of medial osteophytes reveals the true floor of the acetabulum. A drill can also be used to gain an understanding of the remaining medial wall with a depth gage to be sure not to go too deep. The surgeon can use the table, floor, room, and handle to get the correct cup alignment and anatomic landmarks. In the setting of the dysplastic hip, having a firm understanding of the patient's anatomy keeps the center of rotation low, optimizing hip biomechanics.

**Figure 3:**
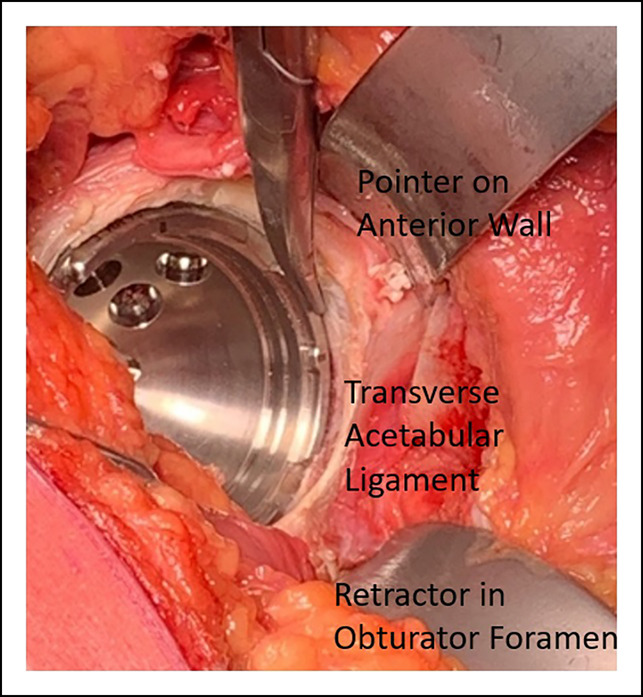
Photograph of cup alignment showing adequate anterior coverage to reduce the risk of psoas impingement.

The psoas recess and transacetabular ligament (TAL) (Figure 3) act as guides in appropriate placement of the acetabular implant. The psoas recess is an anatomic depression on the anterior acetabular ridge where the iliopsoas tendon traverses across the hip joint. The TAL (Figure 3) is a soft-tissue structure part of the acetabular labrum; it crosses across the acetabular notch and prevents inferior migration of the femoral head. Given its constant location within the acetabulum, there has been considerable interest in using it as a guide for cup placement during THA.^20^ In hips without notable dysplasia, one can align the inferomedial aspect of the cup with the TAL to guide the version and inclination. In dysplastic hips, following this landmark has led greater variability in cup version and may not be as useful, hence the need for multiple intraoperative checks.^20^

Maintaining 70% coverage of the shell by the acetabulum and assessing the relationship of the cup to the psoas recess and TAL provide data to get the cup in the desired position. Ensuring the cup is fully seated against the medial wall of the acetabulum is also done at this point. The cup usually sits flush to slightly “tuck in” to the anterior wall and flush to slightly prominent on the posterior wall (Figure 3). A combination of these quick intraoperative checks has led to a reproducible cup position using consistent anatomic landmarks.

### Spinopelvic Parameters

In recent years, there has been great interest in spinopelvic parameters and how they relate to THA. Moving from a standing to seated position causes a posterior tilt to the pelvis, allowing the acetabulum to open to allow for clearance of the hip and increased posterior coverage for stability.^21^ This physiologic motion is impaired in the setting of lumbar spine pathology. While the general population has a dislocation risk of 2% to 5%, those with a history of lumbar spinal fusion are at a risk of 8% to 18%.^21^

Stiff spines are at greatest risk of instability because they are unable to antevert to gain more posterior coverage in a seated position, whereas those with a hypermobile spine become excessively anteverted when seated. With stiff spines, increasing inclination and combined anteversion within a narrower range may prevent impingement.^21^ Hypermobile spines should also have a narrower range of acceptable cup positioning.^21^ There is growing evidence that the use of dual-mobility bearings may reduce the risk of instability in these patients and thus prevent early revision.^22^ Identifying those patients at higher risk of impingement and adjusting implant selection and positioning intraoperatively will reduce the risk of instability and subsequent need for revision.

### Impingement

Psoas impingement is often seen when the anterior acetabular shell sits uncovered and proud of the anterior acetabular wall.^23^ The psoas tendon, which runs along the psoas ridge, can become irritated and attenuated from rubbing over the shell. Patients may present with a snapping sensation or pain with hip motion.^24^ Intraoperatively, ensuring that the acetabular shell is not proud and avoiding excessive dissection around the tendon aids in reducing the risk of injury.^23^ Having greater than 8 mm of acetabular shell uncovered anteriorly is a risk factor for need for revision surgery.^23^ Should there be impingement, conservative treatment may help control the symptoms. In those with refractory symptoms, revision total hip arthroplasty of the acetabulum or a psoas tendon release has been described.^2^

Trochanteric impingement and trochanteric pain are common concerns in the postoperative period after THA. Trochanteric impingement occurs when the trochanter impinges on the acetabulum during a physiologic range of motion. It is estimated that between 3% and 17% of patients will experience some form of this pain, regardless of the approach.^25^ While it is seen more commonly in the posterior approach, it is also seen in direct anterior approaches.^25^ This pain interferes with all facets of postoperative recovery and affects patient-reported outcomes.^25,26^ Failure to recreate sufficient offset and inappropriately shortening the limb increase the risk of trochanteric impingement.^26^ A minimum of 10 mm distance between the medial border of the greater trochanter and the lateral edge of the acetabulum will reduce the risk of trochanteric impingement.^26^ Trochanteric impingement may be managed nonsurgically, although revision may be necessary to treat instability.^2,26^

Liner selection affects impingement in THA. The use of offset liners and lipped liners reduces range of motion. The subsequent impingement of the femoral implant on the liner can lead to instability.^27^

### Restoring Femoral Offset

**Figure 4:**
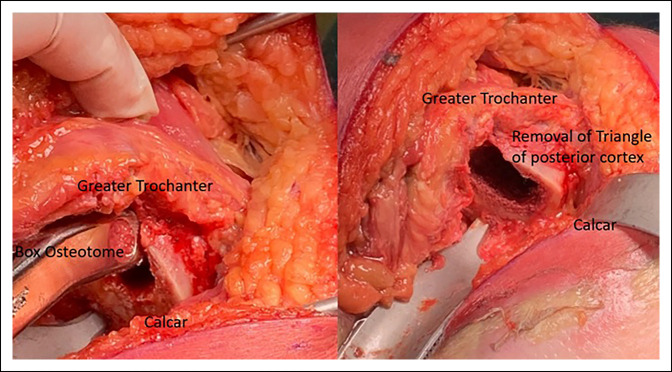
Photograph showing the broaching start point at the piriformis fossa that will leave a small “V” shape of the cortical bone entry point.

In hips with notable trochanteric overhang, a burr may be used to precisely remove the bone after soft-tissue débridement. The piriformis fossa is a constant landmark that may be used when opening the femoral canal. Placing the boxed osteotome at the posterolateral corner of the femoral neck cut at the piriformis fossa and aiming down the shaft of the femur will provide the appropriate start point. There should be a small “V” shaped notch (Figure 4) at the level of the piriformis fossa. The surgeon can ensure appropriate lateralization by placing the tip of the “Canal Finder” broach against the lateral cortex before raising their hand and aiming down the canal. These steps are particularly useful when conducting broaching for a single taper, medial/lateral type 1 noncemented stem design where these single-wedge taper stems fill the proximal femur in the coronal plane and, thus, rely on appropriately placed broaches to maintain rotational stability and attain adequate fill.^28^

Intraoperative femur fractures can occur during broaching or with final prosthesis implantation.^29,30^ Female sex, advanced age, and decreased bone mineral density have been identified as patient risk factors.^30^ Using noncemented “fit-and-fill” ML type 2 stems is an implant-specific risk factor.^30^ The direct anterior approach does pose an increased risk of proximal femur fracture of 5.8%.^31^ This risk is greatest during femoral elevation when the greater trochanter can get hung up on the acetabulum.^31^ For those who are at risk of intraoperative fracture, implant selection can reduce the risk of fracture.^30,32^

Regarding femoral implants, the femoral stem should fill the canal. The prosthesis should rest at the level of the neck cut on the calcar. The prosthesis should be both axially and rotationally sound before leaving the operating room. Stable implants reduce micromotion, early loosening, and failure.^3^ In those with tight distal canals, flexible reamers and intraoperative fluoroscopy can be used to safely open the canal and create space to broach. In those with unstable wedge stems in the setting of poor metaphyseal bone, conversion to cemented prosthesis and a double taper “fit-and-fill” type stem are two options to create femoral stem stability.^28^

### Aseptic Loosening and Osteolysis

Aseptic loosening is the most common indication for late revision THA.^3^ This phenomenon can occur because of inadequate fixation of implants, by cyclic loading, or by particle-induced osteolysis around implants.^3,33^ The risk of aseptic loosening increases approximately 1% annually for THA.^3^

Cyclic loading occurs with every day weight bearing. Implant and bearing materials are important because a prosthesis that allows for interdigitation and bearing materials that are long-lasting and resistant to this cyclic loading will extend the lifetime of the implant and prevent premature failure. Regarding noncemented prosthesis, both ingrowth and ongrowth surfaces of the prosthesis help stabilize the implant. The optimal pore size of 50 to 300 μm and porosity of 40% to 50% confer long-term stability without increasing risk of failure of the prosthesis.^34^

Particulate-induced osteolysis around the implant occurs with all current bearing types—metal-on-metal (MoM), metal-on-polyethylene, ceramic-on-polyethylene (CoP), and ceramic-on-ceramic. The size, type, immunogenicity, and rate of accumulation of these particulates vary. These variables influence the degree of osteolysis and subsequent loosening that may occur. While MoM bearings created less debris, the particles were smaller in size causing ATLRs.^33,35^ Cobalt-chromium has the highest association with ATLR, although it is seen with other bearing surfaces.^3,33^ MoM hips are also associated with systematic metal toxicities and local abductor insufficiency from local toxicity of the metals.^33,35^ Other bearing choices include a combination of metal, highly cross-linked ultrahigh molecular-weight polyethylene, and ceramic.^33^ Ceramic-on-ceramic bearings have the best wear rates to date, but they are associated with squeaking and catastrophic failure.^3^ Polyethylene liners do not have the risk of catastrophic failure seen with ceramic bearings but carry a higher risk of osteolysis.^33^ This risk has decreased with the advent of highly cross-linked polyethylene bearings. Currently, most implants in the United States are ceramic or metal on polyethylene.

### Neck Cut/Leg Length Inequality

There are several intraoperative landmarks used, along with the preoperative template, to help the surgeon make an appropriate neck cut. Typically, the neck cut is 1 to 2 cm over the lesser trochanter, but this can vary based on the neck-shaft angle and the length of the femoral neck and should be checked on the preoperative template. Tools used to make an appropriate neck cut include a preoperative femoral stem template, the head and neck trial for the specific implant, and the templated distance from the lesser trochanter. Placing the femoral stem template against the proximal femur is used to check the proposed neck cut.^36^ When placing a head and neck trial at the level of the planned cut, its center of rotation should align with that of the native hip.^5,36^ The planned neck cut should also be near the level of the posterior capsular reflection on the neck and exit laterally at the piriformis fossa consistently.^37^ Marking the prospective neck cut also reduces the risk of an errant cut that would cause the need for repeat cutting or loss of surgical time by need for the calcar planar.

Using three separate intraoperative checks allows us to better recreate our template and avoid loss of efficiency by avoiding recuts or calcar planning. It also allows execution of the planned restoration of offset and leg length. An example includes the dysplastic hip with a notable valgus neck requiring a longer cut, which can be confirmed by checking the center of rotation of the femoral head and lesser trochanter. When making the femoral neck cut, a saw is used to make the initial cut, followed by a counter cut under the greater trochanter at the piriformis fossa, which can be accomplished using a saw or osteotome.

**Figure 5:**
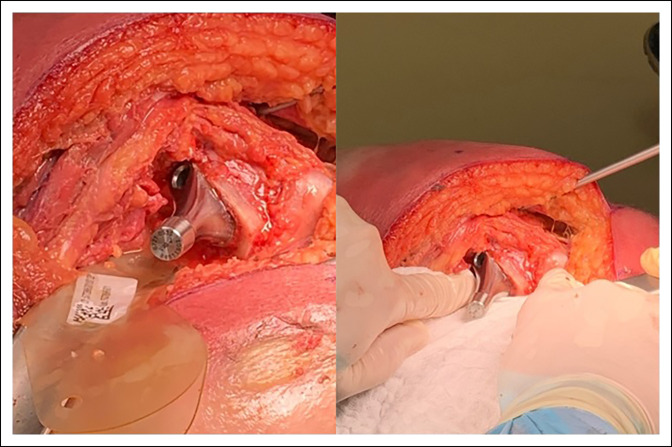
Photographs showing offset check with piriformis. With trial implants placed and the hip reduced, the tension of the piriformis tendon when it is pulled to its insertion on the greater trochanter is used as an intraoperative check for adequate offset.

Assessing leg lengths may be done with trial prostheses. Assessing the relationship between the inferior poles of the patellae and heels are ways to grossly assess the length. The surgeon can measure the length of the implant shoulder to the greater trochanter and compare it with the preoperative template. The trunnion should sit at the level of the tip of the greater trochanter as well. Adequate soft-tissue tension of the abductor muscles with the hip reduced and when the piriformis tendon is placed against its insertion on the greater trochanter (Figure 5) provide another check to adequate length and offset of the construct and reducing the risk of impingement.

### Prosthesis Protection

**Figure 6:**
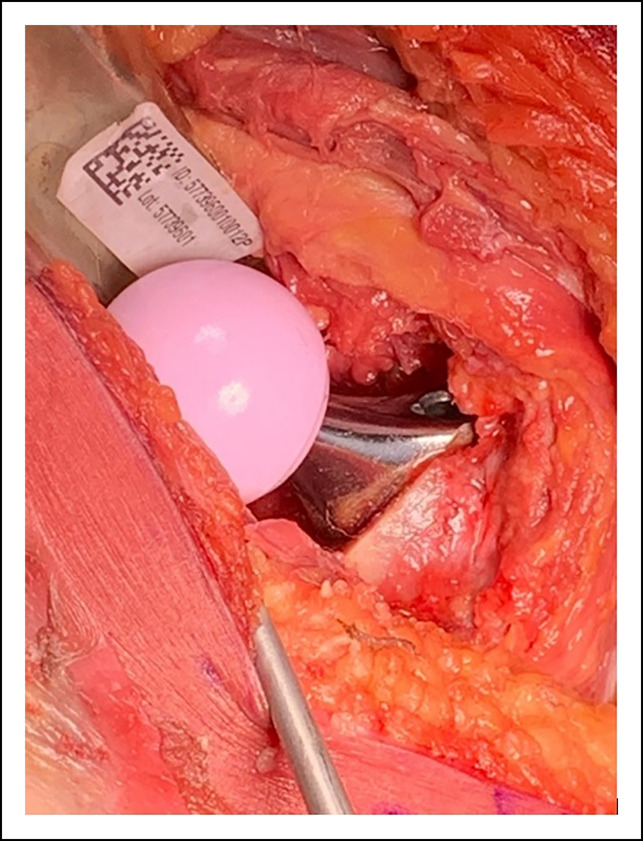
Photograph showing combined version check using the Ranawat sign.

Trialing allows for any changes to offset; leg length or cup position is made to customize the construct to the needs of the patient. Once the hip is reduced, the relationship in version and alignment between the implants is checked utilizing the Ranawat Sign.^38^ (Figure 6). With the hip in extension and internally rotated to 45°, the equator of the trial head should sit parallel to the equator of the liner and acetabulum. If these are not parallel, the surgeon would be able to adjust the acetabular implant before inserting a pelvic screw for additional fixation. Adducting the leg in the “sleeper position” and flexing the hip to 90° and then internally rotating to see the stable arc of motion are other tests conducted to assess for a physiologic stable arc of motion. Lacking 60° of internal rotation increases the risk of dislocation postoperatively.^39^ Using trial implants during stability testing reduces the risk of damage to the final prosthesis, thus reducing the risk of early failure.

**Figure 7:**
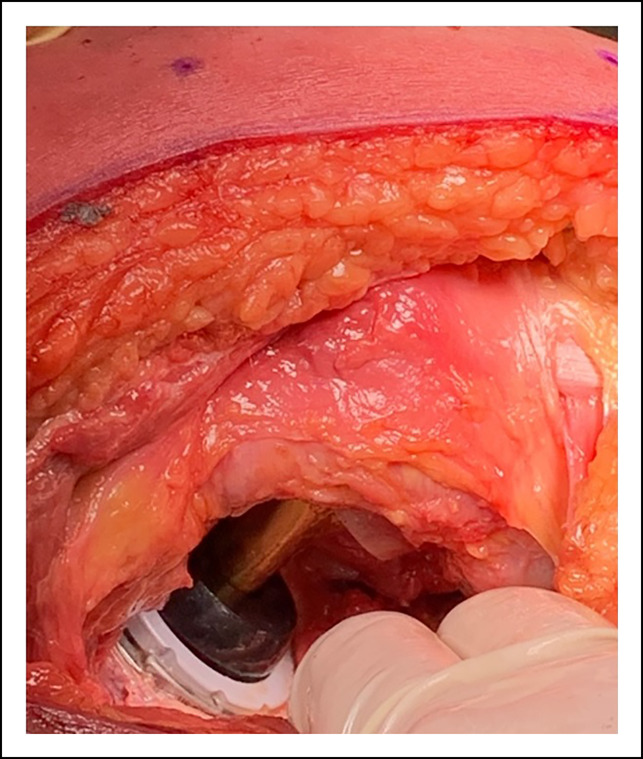
Photograph showing a ceramic-head/metal-head sleeve. Using the femoral stem sleeve over the retractors, the head is protected from stripe wear when removing retractors.

When implanting the femoral implant, the plastic liner of the femoral stem packaging is placed over the calcar retractor, and the stem is then inserted. This plastic piece prevents errant damage should the retractor slip and scratch the final prosthesis and reduce the risk of stripe wear once the head is affected (Figure 7). Trialing with plastic heads is done next using the same landmark checks and range-of-motion assessments. Stripe wear and third-body wear can occur during the dislocation and reduction maneuvers.^40^ Damage to the trunnion has also been implicated as a cause of mechanically assisted crevice corrosion (MACC).^40–42^ Lack of careful retraction and instruments contacting the trunion could cause damage and potentially contribute to MACC. When mating the head to taper, one strike with a force of 14 kN conferred the same stability as multiple strikes. Insufficient strikes may lead to inadequate head-taper mating and, thus, MACC.^42^

In modern arthroplasty, the taper-head junction is modular. With this modularity, there is the risk of MACC.^40^ The micromotion between two bearing surfaces leads to degradation of the implants and failure. This occurs most commonly when a cobalt-chromium head or sleeve is used in conjunction with a different metal, usually titanium. However, MACC has been seen with other bearing surfaces. The length and width of the head-neck taper may play a role as the increased surface area may contribute to greater corrosion. Several authors have proposed that a shorter or narrower taper increases the micromotion of the head and neck, leading to greater corrosion.

**Figure 8:**
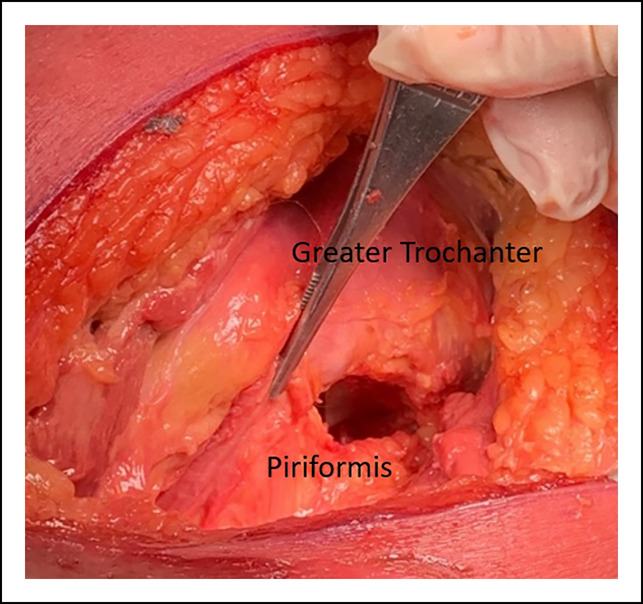
Photograph showing clean trunnion and calcar retractor protected with the femoral stem plastic sleeve. Keeping the taper clean and protected reduces the risk of MACC. MACC = mechanically assisted crevice corrosion

Minimizing the damage to the femoral prosthesis reduces the risk of MACC and ALTR. Keeping the trunnion and inner sleeve of the head clean and dry prevents debris from occurring in the head-taper interface and allows for a better fit (Figure 8). This reduces the risk of increased micromotion, debris formation, and failure. Similarly, avoiding repeated dislocations and reductions with final implants reduces the risk of damage to the bearing surfaces. With the final implants in place, the wound is then thoroughly irrigated and closed.

## Summary and Recommendations

There are many tools at the surgeon's disposal to prevent common causes of early revision intraoperatively. Although complication rates remain low for THA, understanding the possible pitfalls of this procedure and how to avoid them can help both patient and surgeon have a successful surgery.

Preoperative patient optimization and preoperative templating help the surgeon plan for patient-specific needs during the case. Thoughtful selection of implants and bearing types reduce the risk of osteolysis, aseptic loosening, and ALTR. Identification of anatomic landmarks and multiple alignment and stability checks provide the surgeon with multiple points of data confirming appropriate patient-specific alignment. The prosthesis should be protected during insertion to limit corrosion and soft tissue reactions. THA is considered to be the surgical procedure of the 20th century.^1^ As our population ages, the number of THA will continue to rise.^1^ As such, the need for revision surgery will correspondingly increase. Understanding common issues encountered in a primary THA and knowing techniques to avoid causing premature failure and need for revision surgery will afford a greater quality of life to our patients.
